# Empowering University Employees Through Support for Family Caregiving

**DOI:** 10.1093/geroni/igaf122.1270

**Published:** 2025-12-31

**Authors:** Katherina Nikzad-Terhune, Allyson Graf, Lydia Manning

**Affiliations:** Northern Kentucky University, Highland Heights, Kentucky, United States; Northern Kentucky University, Highland Heights, Kentucky, United States; Scripps Gerontology Center, Oxford, Ohio, United States

## Abstract

Informal caregiving continues to increase, and caregivers are becoming more diverse, prompting continued efforts to examine the impact of informal caregiving. Focusing on caregivers in the higher education landscape is beginning to gain more traction as the impact of population aging and caregiver diversity becomes more evident. Supporting faculty and staff caregivers in higher education is essential for fostering a more inclusive and equitable environment, allowing them to more effectively balance professional and caregiving responsibilities. The evidence-based age-inclusivity domains of higher education (AIDHE) model guides institutions of higher education in assessing age-inclusive practices that impact faculty and staff across seven domains of institutional function. Data from a campus-wide caregiving study revealed that nearly one in four employees (24.6%) identified as caregivers, most commonly to aging parents and spouses. Stress from caregiving, work conflicts, and care for others in the household were cited as limiting caregiving ability. Approximately half (47.5%) experienced low quality of life due to lacking caregiver support. The majority (58.5%) were unaware of support services for caregiving. The experiences of faculty and staff caregivers highlight the need for better recognition and support services from institutions of higher education. Based on study findings and framed through the lens of the AIDHE model, recommendations will be made for universities to capitalize on pre-existing campus programs and resources, and to develop new age-inclusive programs and practices to effectively respond to the needs of faculty and staff caregivers.

